# Cost-effectiveness of a technology-assisted peer-delivered perinatal mental health intervention in Pakistan: an economic evaluation using trial evidence

**DOI:** 10.1136/bmjgh-2025-020833

**Published:** 2025-11-13

**Authors:** Naomi Kate Gibbs, Tao Chen, Abid Malik, Huma Nazir, Anum Nisar, Ahmed Waqas, Najia Atif, Duolao Wang, Atif Rahman, Siham Sikander, Simon Mark Walker

**Affiliations:** 1 Centre for Health Economics, University of York, York, UK; 2 Liverpool School of Tropical Medicine, Liverpool, UK; 3 Health Services Academy, Islamabad, Pakistan; 4 Human Development Research Foundation, Rawalpindi, Pakistan; 5 University of Liverpool, Liverpool, UK; 6 Department of Psychological Sciences, University of Liverpool, Liverpool, UK

**Keywords:** Health economics, Maternal health

## Abstract

**Introduction:**

Perinatal depression in low- and middle-income countries is a global health concern. Interventions to support women suffering from perinatal depression using mental health specialists, such as the WHO Thinking Healthy Programme (WHO-THP), are established but may not be scalable in resource-constrained settings. The technology-assisted peer-delivered THP (THP-TAP) has been developed as a potential solution to deliver an intervention at scale. This study assesses whether the THP-TAP is cost-effective compared with the WHO-THP in Pakistan.

**Method:**

Using data for 980 pregnant women from a cluster-randomised non-inferiority trial in Pakistan, we conducted a within-trial cost-effectiveness analysis of THP-TAP compared with WHO-THP. Health outcomes are quality-adjusted life-years (QALY) and costs in US$ (2022). Costs collected included intervention delivery costs and wider healthcare resource use costs. The trial intervention delivery costs were adapted to ‘real-world’ intervention delivery costs using evidence and assumptions. Uncertainty was explored through scenario and sensitivity analyses.

**Results:**

During the trial, the mean patient QALYs were 0.683 (0.681, 0.685) for WHO-THP and 0.688 (0.686, 0.690) for THP-TAP, resulting in an incremental increase in QALYs of 0.005 (0.002, 0.008). The mean per patient costs were $279 ($268, $290) for WHO-THP and $227 for THP-TAP ($218, $236), resulting in an incremental cost of −$52 (−67, −$38). The per patient delivery costs were estimated at $44 and $24 in the real-world scenario, whereas in the trial they were $59 and $69, for WHO-THP and THP-TAP, respectively.

THP-TAP is both more effective and less costly than WHO-THP. These results were robust when considering parameter uncertainty and across various scenarios.

**Conclusions:**

Our analysis suggests that THP-TAP could represent a scalable, health-improving and cost-saving intervention to support those with perinatal depression, when compared with WHO-THP.

**WHAT IS ALREADY KNOWN ON THIS TOPIC**

- Perinatal depression is a global health concern with around one in four pregnant women in low- and middle-income countries affected. The Thinking Healthy Programme developed by the WHO (WHO-THP) is effective in supporting women with perinatal depression in low-income settings. The recent cluster randomised trial in Pakistan has indicated that delivering the intervention as technology-assisted peer delivery (THP-TAP) was non-inferior to delivering it using trained mental health workers.

**WHAT THIS STUDY ADDS**

- In this study, we present the within-trial cost-effectiveness results of THP-TAP compared with WHO-THP, in Pakistan, to assess whether it improves population health and crucially also provides value for money. Cost-effectiveness analysis of digital mental health interventions is less prevalent in resource-constrained settings, but such interventions have great potential to improve population health where healthcare staff constraints are most severe.

**HOW THIS STUDY MIGHT AFFECT RESEARCH, PRACTICE OR POLICY**

- The evidence suggests that THP-TAP could represent a scalable, health improving and cost-saving intervention to support those with perinatal depression, when compared with WHO-THP.

## Introduction

### Perinatal depression burden

 Perinatal depression is a global health concern with around one in four pregnant women in low- and middle-income countries (LMICs) affected,^1^ where the perinatal period is generally defined as the period from pregnancy to 1 year post birth. In Pakistan, the prevalence of perinatal depression may be higher, with a recent systematic literature review and meta-analysis estimating the prevalence of antenatal depression at 37% (95% CI 30 to 44), while that of postnatal depression was estimated at 30% (95% CI 25 to 36).^2^

Perinatal depression poses significant public health challenges, linked to adverse outcomes in children’s cognitive, socioemotional and physical development.^3 4^ It also perpetuates global health and socioeconomic inequalities. Addressing common perinatal mental disorders, such as postnatal depression, could reduce their substantial societal costs, which in the UK total approximately £8.1 billion annually per birth cohort.^5 6^ Research by Bauer *et al* suggests that improving services for these conditions could cost less than a fifth of the current societal burden, underscoring the need for better interventions and support.

### Health interventions for perinatal depression

Health interventions to support women with perinatal mental health problems have been developed. The Thinking Healthy Programme developed by the WHO (WHO-THP) aimed to reduce perinatal depression in low socioeconomic settings and improve health outcomes in children through the adaptation and integration of cognitive behavioural therapy (CBT) into the routine work of community health workers. Starting from pregnancy and continuing postnatally, participants received around eight sessions of the evidence-based ‘talking therapy’.^7^,^9^ Following concern that this may not be scalable due to budget impact and human resource constraints, in particular the workload of community health workers called lady health workers (LHWs) in the Pakistani setting, an adapted version was created to be delivered by peers.^10^,^12^ To ensure fidelity to the intervention, a further adaptation was developed to support the peers via technological tools. The Technology Assisted Peer delivered THP (THP-TAP) is delivered using a tablet and an application. Peers are lay women, without any formal mental health training, who have shown an interest or desire to help and support other women within their community. This adapted intervention was compared with the original WHO-THP intervention during the non-inferiority trial, and results indicated the THP-TAP to be non-inferior.^13^

Effectiveness evidence must be coupled with an understanding of the cost of the intervention, and how it compares with alternative uses of the same resources, to usefully inform healthcare decisions around resource allocation and prioritisation. Cost-effectiveness analysis provides a framework for presenting this evidence and is well established in healthcare decision making.^14 15^ Two systematic reviews of cost-effectiveness studies of interventions to address perinatal depression and/or anxiety have reported results which demonstrate good value for money but are exclusively drawn from high-income settings.^16 17^ There are a number of examples of cost-effectiveness analysis of digital mental health interventions,^18 19^ but again they are less prevalent in resource-constrained settings, despite such interventions having great potential to improve population health where healthcare staff constraints are most severe. In this study, we aim to present the within trial cost-effectiveness results of THP-TAP compared with WHO-THP in Pakistan to assess whether it improves population health and provides value for money.

## Methods

### Overview

We conducted an economic evaluation of THP-TAP compared with WHO-THP to estimate its value for money and impact on population health. We conducted a within-trial cost-effectiveness analysis and then examined the potential health impacts of scale-up of the interventions given available resources. Health outcomes are presented in quality-adjusted life-years (QALYs), a composite measure of health which includes morbidity and mortality, and costs take a health system perspective (ie, we combine healthcare costs which accrue to both the public and private sector). Cost-effectiveness is presented using incremental cost-effectiveness ratio (ICER) (ratio of the difference in mean costs and mean QALYs) and net health benefits (NHBs) (the health gain from a treatment less the health which could be gained elsewhere in the health system by the use of those resources). To assess value for money, a cost-effectiveness threshold based on an empirical estimate of the marginal cost of producing health in Pakistan is used. Finally, we estimate the health impact of scale-up given the size of the patient population nationally and available resources. We do not discount costs and outcomes as the costs and effects are only considered for the within-trial period. The mean intervention period was 10 months. This consisted of a standard 6-month postnatal period for all women and a mean prenatal period of 4 months as recruitment into the trial could vary between the second and third trimester of pregnancy.

### Technology-assisted peer-delivered perinatal mental health trial

The trial was a cluster-randomised non-inferiority trial of technology-assisted CBT, delivered by peers (THP-TAP), versus standard CBT, delivered by LHWs (WHO-THP), for perinatal depression in Pakistan. Peers were laywomen from the community with no formal healthcare training but with experience of motherhood. It was a stratified cluster randomised controlled trial design with 70 village clusters.^20^ The aim of the trial was to establish non-inferiority, that is, delivering the intervention using peers assisted with tablets was not inferior to delivering the intervention using LHWs.

Participants were consenting women in their second or third trimester of pregnancy, 18 years and over, living in the village clusters and on the registers of the LHWs, with a current major depressive episode assessed using a structured clinical interview. The intervention consists of eight home visits from either a LHW (WHO-THP) or a peer who had been provided a tablet to operate the THP app (THP-TAP). The tablet provided the platform for the cognitive behavioural elements of the intervention using animated avatars of therapists, clients and community members and a narrative storytelling approach which had been coproduced to ensure cultural compatibility. The peer was then able to focus on delivering empathy and support, the social ingredients of the intervention. The app and the peer effectively worked together to deliver the psychological and the social elements of the intervention. For clarity, the LHWs did not use tablets but delivered the CBT intervention themselves. Participants were followed up to 6 months after birth.

### Resource use and costs

We capture the costs of intervention design and delivery as well as mother and child’s wider healthcare resource use.

#### Delivery costs

Intervention delivery costs were reported for both trial arms which included equipment, training, supervision, quality assurance and financial incentives for the peers or LHWs. All unit costs and resource-use estimates were provided by the finance team based in Pakistan who administered the trial.

Two costing analyses were considered for delivery of the intervention, one based on the trial whereby total trial intervention delivery costs were allocated based on number of patients in the trial, and another using an optimised approach reflecting likely real-world use of the intervention reflecting increased throughput and duration over which deliverers could provide the intervention to new women. We use the estimated birth rate of 124 per 1000 as a proxy for pregnancies (this will be an underestimate due to miscarriage) (Government of Pakistan, 2022). Prevalence of depression is assumed to be 32% of pregnant women; this is a midpoint between two recent estimates, 27% and 37% (Atif *et al*, and Khan *et al*). The population of an area covered by peers is assumed to be 1000, therefore each peer would see 40 women, assuming all depressed pregnant women were seen. The population in Pakistan in 2024 is 245.2 million (United Nations Population Fund, 2024).

A list of the data and assumptions underpinning both approaches is included (online supplemental table 1). Our base case analysis used the expected real-world delivery costs, while trial delivery costs are presented as a scenario.

#### Healthcare resource use

We capture the cost of other healthcare resource use during the trial period. The resource use was collected from patients using a modified Client Services Receipt Inventory (CSRI), a series of questions which ask patients about the number of contacts they have had with various healthcare services. The CSRI has been used in previous trials in Pakistan and cross-culturally validated.^21^ The CSRI was administered face to face at baseline, 3 months and 6 months after birth. Healthcare resource use is categorised as contact with health and social care professionals, hospital inpatient services and paediatric services. Unit cost estimates for each service are based on internal financial records and estimates, for both the private and public sector. Due to the mixed healthcare system, costs reflect those falling on public or private sectors/providers. The resource use and unit costs are combined to report total costs for the mother only in the base case and for the mother-child dyad as a scenario.

Costs are converted into US$ from Pakistani Rupees using the average exchange rate for 2022.^22^ Unit costs were inflated where necessary by increasing prices to June 2022 from the time period they were reported using the Pakistani consumer price index inflation.^23^

The base case analysis takes a healthcare perspective, which combines the costs accrued by the public and private sector. Out of pocket (OOP) costs paid by the individual are reported separately.

For the base case, we exclude paediatric costs and only include healthcare resource use costs to the mother, to coincide with the health outcomes, which only relate to the mother.

### Health outcomes

The primary health outcomes were QALYs, a composite measure of health which captures both morbidity and mortality calculated by combining a health-related quality of life (HRQoL) score (where zero is equivalent to death and one is equivalent to full health) with the time spent at that level of health. The HRQoL score was based on individuals’ responses from the EuroQol 5 Dimension 3 Level (EQ-5D-3L) questionnaire measured at baseline and 3 months postnatal (which defines the health state) with scores based on the Pakistan value set.^24^ Linear interpolation was used to capture change in health between baseline and 3-month postnatal follow-up, and the 3-month postnatal HRQoL was assumed to last until the 6-month postnatal follow-up. In response to this limitation, we include a scenario whereby both groups achieve the same HRQoL (that of the THP-TAP at 3 months) by 6 months. This is a conservative approach which implies that treatment benefit, in terms of HRQoL, completely wanes by the 6-month follow-up. QALYs were calculated based on the average time between follow-up points to remove variation in follow-up length impacting results.

Health outcomes to the children are not included in this cost-effectiveness analysis.

### Analysis

Cost-effectiveness results were calculated over the patient within trial time-horizon (from baseline to 6 months postnatal^1^) with outcomes in QALYs and costs from the health system perspective.

Regression analysis was used to estimate the impacts of intervention on costs and QALYs while controlling for patient covariates. Ordinary least squares regression was used to estimate QALYs. Generalised linear models with log link and gamma were used for the cost data to accommodate the non-negative and skewed data. Baseline variables included in both models were treatment allocation, age, whether the woman had given birth before (including stillbirths) and whether the reported monthly income was below Pakistani Rupee (PKR) 25 000. Baseline HRQoL scores were included as covariates in the QALY regression analysis, while baseline healthcare resource use costs by category were included in the cost regression analysis. The inclusion of paediatric costs is explored as a scenario analysis.

Multiple imputation by chained equations with predictive mean matching was used to impute missing cost and HRQoL for individual patients due to loss to follow-up. The number of imputed datasets was set equal to the percentage of missing data,^25^ assuming data are missing at random. To ensure that all available data are used, we imputed values by healthcare category for costs (ie, health and social care contacts, inpatient cost and paediatric costs) split by public, private and OOP. Outputs which support the reliability of the multiple imputation are given in the online supplemental materials.

Cost-effectiveness results are presented using ICERs and NHB. A cost-effectiveness threshold estimated for Pakistan of $191 or PKR 39 130 (using the 2022 exchange rate of 1:204.87^22^) per QALY is used.^26^ This figure is an empirical estimate of the marginal cost, in Pakistan, of producing one additional unit of health or QALY in this case, denoted as ‘k’. We also present incremental NHB, which is the difference between the change in QALYs from the intervention less the health which is forgone elsewhere by not using the resources for alternative health generating activities (estimated by converting the incremental costs into health using the cost-effectiveness threshold). A positive incremental NHB indicates a positive population level health gain as you are gaining more health than you lose. We will present the costs, QALYs, ICER and NHB as a point estimate. To capture parameter uncertainty, we also conduct probabilistic sensitivity analysis. This provides a 95% credible interval computed from 1000 simulations assuming multivariate normality of the coefficients from the regression equations to estimate uncertainty while capturing the correlation between variables used in the cost and QALY regression models.^27^

#### Scenario analysis

We consider four alternative scenarios. First, paediatric costs were included. second, using delivery costs based directly on the trial rather than using the optimised assumptions. Third, using a per protocol analysis which only includes patients who completed the planned treatment. Lastly, assuming the 6-month HRQoL is the same for both arms of the trial and is that of the 3-month THP-TAP.

#### Patient and public involvement

The THP-TAP intervention was developed with substantial input from local stakeholders, including an expert-by-experience as a co-investigator, ensuring patient and public involvement in the design and adaptation of the intervention. All efforts were made to ensure that the research did not result in stigmatisation, incrimination or discrimination against participants.^13^

## Results

### Patient population

There were 980 patients recruited to the trial, of whom 487 received THP-TAP and 493 received WHO-THP. The mean age was 27 (SD=5), mean parity was 1.62 (SD=1.48) and mean monthly income was PKR 32 028 (SD PKR 48 653).

### Missing data

At the 3-month postnatal data collection, 12% of patients were lost to follow-up for THP-TAP and 15% for WHO-THP, increasing to 13% and 15%, respectively, at the 6month postnatal follow-up. Further details on missing data and participant characteristics are reported in the clinical trial results paper.^13^

### Resource use and costs

The per patient delivery costs were $44 and $24 for WHO-THP and THP-TAP, respectively, assuming the intervention resources are optimised with wider roll out (table 1). The within-trial per patient delivery costs were $59 and $69 for WHO-THP and THP-TAP. The delivery unit costs and summary costs are provided (online supplemental tables 2 and 3). The healthcare resource use during the trial was broadly similar across arms with inpatient services representing the largest share of costs. Detailed resource use and healthcare unit costs are provided in the online supplemental tables 4 and 5.

**Table 1:** Within-trial per patient costs by intervention, missing values have been imputed

	WHO-THP Mean (SD)	THP-TAP Mean (SD)
Delivery costs		
Per patient within trial	$59	$69
Per patient real-world	$44	$24
Healthcare resource use costs		
Health and social care professional contacts	$9 ($13)	$7 ($10)
Inpatient services	$238 ($449)	$199 ($428)
Paediatric services	$78 ($272)	$60 ($231)
OOP costs		
Health and social care professionals	$21 ($32)	$17 ($27)

Costs in US$ 2022 prices.

Converted from PKR using the 2022 exchange rate of 1:204.87.^22^

THP-TAP, technology-assisted peer-delivered Thinking Healthy Programme; WHO-THP, WHO Thinking Healthy Programme.

### Health outcomes

HRQoL scores of both groups increased from baseline (0.698/0.693, WHO-THP/THP-TAP) to 3 months postnatal (0.909/0.919 WHO-THP/THP-TAP). A table comparing results, with and without multiple imputation, is provided (online supplemental table 6). EQ5D-3L scores improved across all five health domains, with the greatest improvement in the anxiety and depression domain (online supplemental table 7). The mean time in the trial was 10 months, consisting of 4 months prenatal and 6 months postnatal. This was used as the analytical timeline to allow for consistent QALY estimates across groups. Controlling for covariates, the mean patient QALYs, generated over these 10 months, were 0.683 (0.681, 0.685) for WHO-THP and 0.688 (0.686, 0.690) for THP-TAP, resulting in an incremental increase in QALYs of 0.005 (0.002, 0.008).

### Cost-effectiveness results and scenario analysis

In the base case scenario, THP-TAP is both more effective (0.005 incremental QALYs) and less costly (−US$52) and therefore ‘dominates’ WHO-THP (table 2). Full details of the regression models are available in the online supplemental tables 8 and 9. The NHB estimate is 0.278 QALYs, implying THP-TAP will increase population health, compared with WHO-THP. This is higher than the incremental QALYs (0.005) as the resources saved can be released and used for other productive healthcare, increasing population health. The THP-TAP remains dominant in all three scenario analyses.

**Table 2:** Probabilistic cost-effectiveness analysis results for THP-TAP versus WHO-THP for baseline and scenarios

	Costs	QALYs	Incremental costs	Incremental QALYs	ICER	NHB (k = $191)	Probability of being cost-effective
Mean (95% credible Intervals)							
Base case							
WHO-THP	$279 ($268, $290)	0.683 (0.681, 0.685)					
THP-TAP	$227 ($218, $236)	0.688 (0.686, 0.689)	−$52 (−$67 to −$38)	0.005 (0.002, 0.008)	Dominant	0.278 (0.204, 0.357)	100%
Healthcare perspective including paediatric costs							
WHO-THP	$357 ($344, $370)	0.683 (0.681, 0.685)					
THP-TAP	$287 ($277, $298)	0.688 (0.686, 0.69)	−$70 (−$87 to −$54)	0.005 (0.002, 0.008)	Dominant	0.369 (0.287, 0.461)	100%
Delivery costs as per trial							
WHO-THP	$292 ($282, $303)	0.683 (0.681, 0.685)					
THP-TAP	$272 ($262, $281)	0.688 (0.686, 0.69)	−$21 (−$35 to −$7)	0.005 (0.002, 0.008)	Dominant	0.112 (0.039, 0.19)	99.9%
Per protocol analysis							
WHO-THP	$301 ($288, $314)	0.697 (0.696, 0.699)					
THP-TAP	$241 ($230, $251)	0.701 (0.7, 0.703)	−$60 (−$77 to −$44)	0.004 (0.002, 0.006)	Dominant	0.319 (0.236, 0.408)	100%
HRQoL equal in both arms by 6 months							
WHO-THP	$279 ($268, $290)	0.684 (0.686, 0.689)					
THP-TAP	$227 ($218, $236)	0.688 (0.686, 0.689)	−$52 (−$67 to −$38)	0.004 (0.001, 0.006)	Dominant	0.277 (0.203, 0.356)	100%

ICER, incremental cost-effectiveness ratio; NHB, net health benefit; QALYs, quality-adjusted life-years; THP-TAP, technology-assisted peer-delivered Thinking Healthy Programme; WHO-THP, WHO Thinking Healthy Programme.

**Figure 1:**
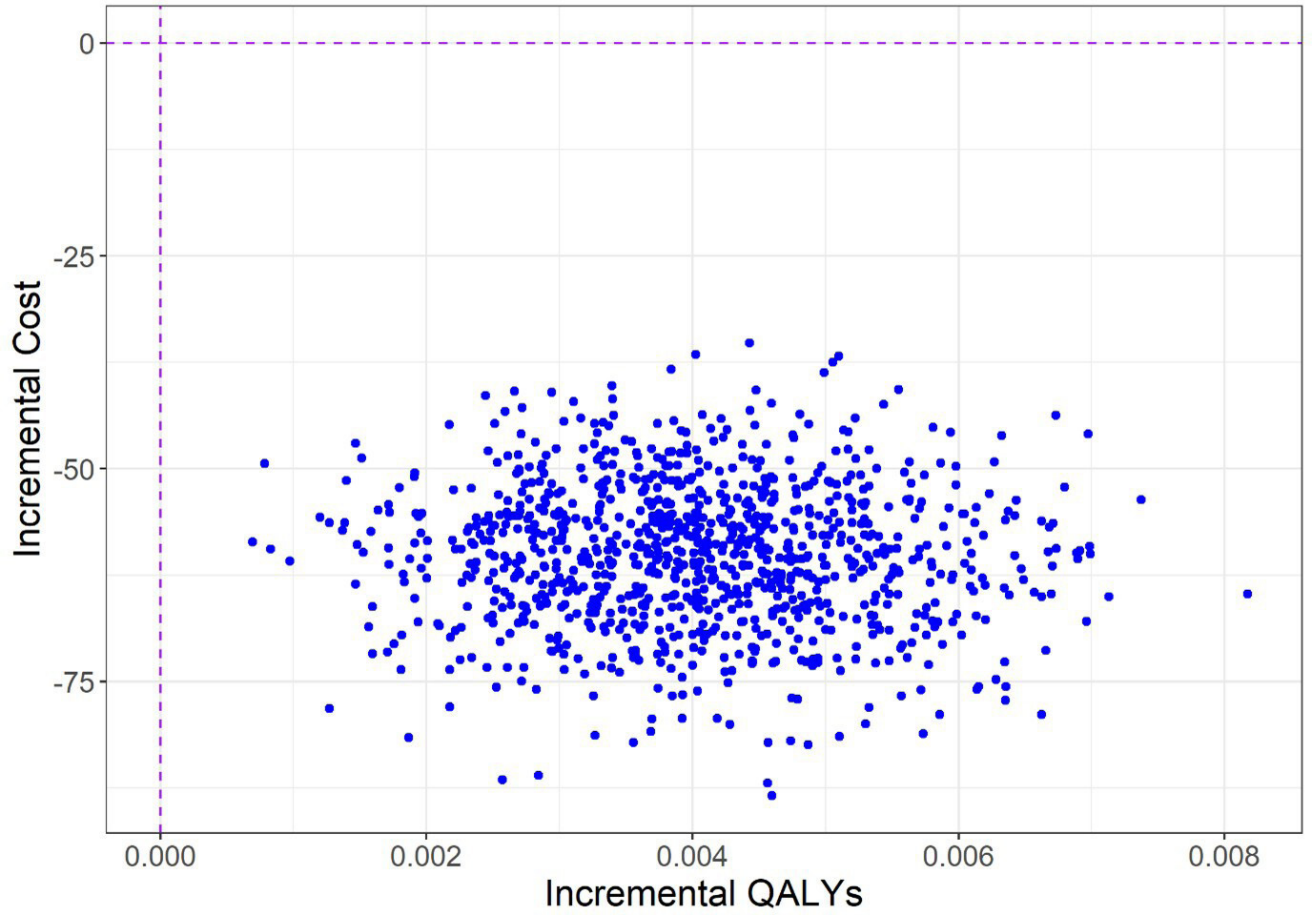
Cost-effectiveness plane comparing THP-TAP with WHO-THP. QUALYs, quality-adjusted life-years; THP-TAP, technology-assisted peer-delivered Thinking Healthy Programme; WHO, WHO Thinking Healthy Programme.

The incremental costs and QALYs of the 1000 simulations from the probabilistic analysis are plotted on the cost-effectiveness plane (figure 1). All but one simulation lies in the southeast quadrant, suggesting THP-TAP is dominant (ie, health improving and cost-saving) and the probability of being cost-effective is nearly 100% at all cost-effectiveness thresholds. In all four of the scenarios the THP-TAP remains cost-effective in comparison with WHO-THP.

Using the assumptions related to scale-up, we estimate THP-TAP would require a workforce of 245 200 peers to deliver the intervention to 9 729 536 perinatally depressed pregnant women per year. This would generate a population level NHB of 2 704 811, in comparison with delivering WHO-THP, per year.

## Discussion

Our analysis suggests that THP-TAP is both more effective and less costly than WHO-THP. We have estimated the delivery cost of THP-TAP, should the intervention be scaled up to a national level, to be US$20 dollars less per patient than WHO-THP; this is primarily due to the ability to share technology across patients and human resource costs being lower from using peers rather than LHW. Our results are robust to alternative scenarios and parameter uncertainty. Cost-effectiveness studies to inform health decision making are sparse in Pakistan; therefore, our study makes an important contribution to the literature. We also provide a set of unit costs for healthcare resource use which can support other health intervention studies in the region. Our estimates do not include the one-off cost of designing the app ($147 793) as our analysis aims to inform the wider roll-out of this intervention in Pakistan and therefore development costs have been excluded. Should another country be interested in developing a similar app and rolling it out, these costs should be considered in the decision-making process.

The finding that participants receiving THP-TAP received an increase in QALYs relative to those receiving WHO-THP is notable, although the benefit is small and uncertain. There are potential mechanisms by which this increase may have occurred. The THP-TAP intervention was co-produced and delivered using peers and women with lived experience of perinatal depression. It is possible that the peers were better able to relate to the women in the trial and were also better placed to help find solutions to their everyday problems. If so, it may be that the social support element of the intervention is more effectively delivered by peers than the LHWs.^28 29^ In addition, in THP-TAP, each therapy session was directed entirely by the virtual therapist, ensuring that the core therapeutic components were delivered consistently and at the intended dosage, which may have increased effectiveness relative to WHO-THP.

Despite increasing evidence for cost-effectiveness of digital health interventions, most studies are conducted in high resource settings.^19^ Digital interventions have the potential to be more scalable as they require less human resource, such as LHWs. In Pakistan, LHWs currently have a very high workload, layering another intervention on would displace alternative important healthcare. In cost-effectiveness, we account for this displacement using the estimate of the marginal productivity of the health sector, but there is considerable uncertainty around these figures. Methods to account for the mix of private and public healthcare have not yet been fully developed. Given THP-TAP dominates WHO-THP, these estimates are less critical to this specific decision but remain an important question for future cost-effectiveness studies in the country and the region.

There are several limitations to our study. The comparison for our analysis is currently WHO-THP; however, it was considered unlikely by the trial team that this would ever be delivered at scale in Pakistan by LHWs due to their limited capacity. Therefore, a more realistic comparison might be ‘no intervention’. This may substantially change the results of the cost-effectiveness analysis, potentially even changing the decision. However, a recent systematic literature review of digital interventions in mental health found evidence that digital interventions are cost-effective, compared with no intervention and non-therapeutic controls, whereas comparisons with face-to-face therapy or printed manuals remained unclear.^19^

Additional limitations include our analysis only covering the trial period and therefore not accounting for any long-term cost-effectiveness benefit.^30^ Our study is specific to Pakistan and evidence may not be generalisable to other settings. The trial was only powered to detect non-inferiority of THP-TAP versus WHO-THP, so our results suggesting THP-TAP is more effective may not be robust. Patient travel costs were not included in our study as the intervention was delivered at the participants’ homes. If we had collected travel costs of those delivering the intervention, we expect THP-TAP would have lower costs as peers were more closely located to trial participants than LHWs; as such, their exclusion is conservative with regard to incremental costs of the intervention. The estimation of real-world delivery costs required several assumptions; however, these were compiled with local finance and clinical experts and all assumptions are provided for transparency online supplemental table 1. The HRQoL scores were not collected at 6 months postnatal, which would add greater accuracy to our estimations.

Despite these limitations, our results indicate that the technology-assisted peer-delivered approach may be a cost-effective method of delivering psychosocial interventions for common mental disorders. Effective and cost-effective interventions for perinatal depression are likely to reduce intergenerational disadvantage and make a compelling policy case for scale-up in LMICs where the burden from the condition is the greatest.

## Conclusion

Our analysis suggests that THP-TAP could represent a scalable and cost-saving intervention to support those with perinatal depression, when compared with WHO-THP. Further research comparing it with a ‘no intervention’ scenario would be beneficial.

## Supplementary material

10.1136/bmjgh-2025-020833 online supplemental file 1

## Data Availability

No data are available.
