# Supplementary material for: Cost-effectiveness of a technology-assisted peer-delivered perinatal mental health intervention in Pakistan: an economic evaluation using trial evidence
Source: BMJ Glob Health. 2025 Nov 13;10(11):e020833. doi: 10.1136/bmjgh-2025-020833 (PMC12625867; doi:10.1136/bmjgh-2025-020833)
Supplement: online supplemental file 1 [file bmjgh-10-11-s001.docx]

# Supplementary material

[Table 1: Assumptions used to estimate the optimised per patient delivery costs 1](#_Toc206600759)

[Table 2: Delivery unit costs 3](#_Toc206600760)

[Table 3: Trial delivery summary costs 4](#_Toc206600761)

[Table 4: Health care resource use during the trial period 5](#_Toc206600762)

[Table 5: Healthcare resource use unit costs 6](#_Toc206600763)

[Table 6: Comparing HRQoL and QALYs with and without multiple imputation 7](#_Toc206600764)

[Table 7: EQ5D-3L percentage shares across domains at each time point by trial arm 7](#_Toc206600765)

[Table 8: Cost model using Gamma gamily and log link, pooled across 15 MI datasets 7](#_Toc206600766)

[Table 9: QALY linear model, pooled across 15 MI datasets 8](#_Toc206600767)

Table 1: Assumptions used to estimate the optimised per patient delivery costs

|  | Real-world scenario | Trial |
| --- | --- | --- |
| Birth rate is assumed to be 124 per 1000 per year | 0.12 | NA |
| Prevalence of depression assumed to be 32% of pregnant women (27% - 37%) | 0.32 | NA |
| Population of an area covered by LHWs | 1700 | NA |
| Population of an area covered by peers | 1000 | NA |
| Depressed pregnant women in LHW (annual) | 67 | NA |
| Depressed pregnant women in peers area (annual) | 40 | NA |
| Workload LHW (annual) assumes all depressed pregnant women in area are seen | 67 | 7 |
| Workload peers (annual) assumes all depressed pregnant women in area are seen | 40 | 6 |
| Remaining career of LHW, therefore does not need retraining (years) | 23.5 | NA |
| Time a peer stays with the programme (years) | 3 | NA |
| Total patients seen by LHW over career | 1585 | NA |
| Total patients seen by a peer, over their time as a peer | 119 | NA |
| Total visits for each patient | 8 | 7 |
| Life of tablet (years) | 3.0 | NA |
| Length of intervention for patient receiving THP-APP (months) | 3 | 3 |
| Patients the server supports in THP-APP | 1000 | 487 |
| Visit incentive for peers and LHWs | PKR 500 | PKR 500 |
| Monitoring visits for peer delivering the intervention (annual) | 2 | 5 |
| Monitoring visits for LHW delivering the intervention (annual) | 5 | 5 |
| Tablet | PKR 50,000 | PKR 48,532 |
| Printing the health calendar, glossy and ring bound (1 per patient) | PKR 3000 | PKR 3000 |
| Printing of supervision materials, reference manual and session log forms (per patient cost) | PKR 287.50 | PKR 287.50 |
| THP-APP training and supervision | |  |
| **Training the peer trainers** |  |  |
| Sessions | 4 | 4 |
| Hours per session | 4 | 4 |
| Specialist trainers | 1 | 2 |
| Peer trainers | 12 | 4 |
| **Training the peers** |  |  |
| Sessions | 4 | 4 |
| Hours per session | 4 | 4 |
| Peer trainers | 1 | 2 |
| Peers | 12 | 8 |
| **Supervision of peer trainers** |  |  |
| Number per year | 12 | 11 |
| Hours per session | 2 | 2 |
| Supervisors (specialist trainers) | 1 | 2 |
| Supervisees (peer trainers) | 12 | 4 |
| **Supervision of peers** |  |  |
| Number per year | 12 | 11 |
| Hours per session | 2 | 2 |
| Supervisors (peer trainers) | 1 | 2 |
| Supervisees (peers) | 12 | 8 |
| WHO-THP training and supervision | |  |
| **Training the national trainers** |  |  |
| Sessions | 5 | 3 |
| Hours per session | 5 | 5 |
| Specialist trainers | 2 | 2 |
| National trainers | 12 | 3 |
| **Training the LHWs** |  |  |
| Sessions | 5 | 5 |
| Hours per session | 5 | 5 |
| National trainers | 2 | 2 |
| LHWs | 12 | 13 |
| **Supervision of national trainers** |  |  |
| Number per year | 12 | 12 |
| Hours per session | 2 | 2 |
| Supervisors (specialist trainers) | 2 | 1 |
| Supervisees (national trainers) | 12 | 3 |
| **Supervision of LHWs** |  |  |
| Number per year | 12 | 11 |
| Hours per session | 2 | 2 |
| Supervisors (national trainers) | 2 | 2 |
| Supervisees (LHWs) | 12 | 13 |

Table 2: Delivery unit costs

| **Category** | **Unit cost**  **2022 PKR** | **Unit cost 2022 USD** |
| --- | --- | --- |
| **THP-TAP** | | |
| Specialist trainers who train the peer trainers for THP-app, hourly rate | PKR 200.00 | $ 0.98 |
| Peer trainers who train the volunteers to deliver the THP-app intervention hourly rate | PKR 100.00 | $ 0.49 |
| Stipend for each visit to a patient received by peer volunteers | PKR 500.00 | $ 2.44 |
| Cost of all the tablets for the THP-app intervention | PKR 2,717,790.36 | $ 13,265.93 |
| Server monthly maintenance cost for the THP-app | PKR 66,397.53 | $ 324.10 |
| **WHO-THP** | | |
| Specialist trainers who train the national trainers for the WHO-THP | PKR 600.00 | $ 2.93 |
| National trainers who train the LHWs in how to deliver the intervention, 250PKR hourly rate | PKR 303.32 | $ 1.48 |
| LHWs allowance for every visit they deliver the intervention | PKR 500.00 | $ 2.44 |
| LHWs allowance for attending the training session | PKR 400.00 | $ 1.95 |
| LHWs allowance for attending a supervision | PKR 1,000.00 | $ 4.88 |
| LHWs hourly rate | PKR 177.08 | $ 0.86 |
| Printing costs for booklets for patients and supervision materials | PKR 1,944,009 | $ 17,766.87 |
| **Both** | | |
| Lunch cost for attending training | PKR 200.00 | $ 0.98 |
| All prices have been adjusted to 2022.  World Bank estimate for average exchange rate in 2022, 1USD to PKR: 204.87 | | |

Table 3: Trial delivery summary costs

| **Summary costs (2022)** | | | | |
| --- | --- | --- | --- | --- |
|  | **Trial costs** | | **Optimised costs if the intervention were to be rolled out** | |
|  | **WHO-THP** | **THP-TAP** | **WHO-THP** | **THP-TAP** |
| Designing the app | - | PKR 30,278,313 | - | - |
|  | - | $ 147,793 | - | - |
| Delivery of the intervention | PKR 5,913,917 | PKR 6,907,341 | - | - |
|  | $ 28,867 | $ 33,716 | - | - |
| Per patient cost of delivering the intervention PKR | PKR 11,996 | PKR 14,183 | PKR 9,057 | PKR 5,013 |
| Per patient cost of delivering the intervention USD | $ 59 | $ 69 | $44 | $24 |
| **Per patient costs by category** | | | | |
|  | **Trial costs** | | **Optimised costs if the intervention were to be rolled out** | |
|  | **WHO-THP** | **THP-TAP** | **WHO-THP** | **THP-TAP** |
| Tablet costs and server maintenance | - | PKR 8,307 | - | PKR 619 |
| Printing costs | PKR 3,943 | - | PKR 3,288 | - |
| Training costs | PKR 480 | PKR 164 | PKR 6 | PKR 10 |
| Supervision costs | PKR 2,451 | PKR 2,218 | PKR 324 | PKR 379 |
| Monitoring costs | PKR 162 | PKR 79 | PKR 22 | PKR 5 |
| Peer/LHW incentives | PKR 4,960 | PKR 3,415 | PKR 5,417 | PKR 4,000 |
| Total | PKR 11,996 | PKR 14,183 | PKR 9,057 | PKR 5,013 |

Table 4: Health care resource use during the trial period

|  | WHO-THP | | | THP-APP | | |
| --- | --- | --- | --- | --- | --- | --- |
| Health and social care professional contacts for the mother, number (percentage which are private) | | | | | | |
|  | T0 (6mth recall) | T1 (6 mth recall) | T2 (3 mth recall) | T0 (6mth recall) | T1 (6 mth recall) | T2 (3 mth recall) |
| Obstetrician | 1441 (33%) | 1446 (22%) | 61 (41%) | 1346 (29%) | 1463 (23%) | 56 (38%) |
| Midwife/Lady Health Visitor | 28 (39%) | 24 (100%) | 4 (100%) | 17 (35%) | 13 (23%) | 6 (50%) |
| Family doctor | 187 (27%) | 162 (30%) | 119 (40%) | 173 (22%) | 120 (19%) | 101 (35%) |
| Nurse/Lady Health Worker | 18 (61%) | 16 (100%) | 12 (25%) | 23 (78%) | 3 (67%) | 7 (71%) |
| Psychiatrist | 10 (20%) | 5 (40%) | 8 (50%) | 0 | 0 | 1 (100%) |
| Psychologist | 8 (0%) | 0 | 3 (0%) | 0 | 0 | 0 |
| Faith healer | 37 (100%) | 9 (100%) | 0 | 9 (100%) | 0 | 0 |
| Home help | 10 (100%) | 6 (100%) | 0 | 16 (100%) | 0 | 1 (100%) |
| Faith healer | 37 (100%) | 9 (100%) | 0 | 9 (100%) | 0 | 0 |
| Traditional healer | 2 (100%) | 0 | 0 | 1 (100%) | 0 | 0 |
| Traditional birth attendant | 2 (50%) | 5 (100%) | 0 | 1 (100%) | 0 | 0 |
| Hospital inpatient services for the mother (days) – all public sector | | | | | | |
|  | T0 (6mth recall) | T1 (6 mth recall) | T2 (3 mth recall) | T0 (6mth recall) | T1 (6 mth recall) | T2 (3 mth recall) |
| Maternity ward | 34 | 733 | 0 | 13 | 644 | 0 |
| Medical ward | 13 | 2 | 4 | 4 | 5 | 0 |
| Psychiatric ward | 5 | 0 | 0 | 0 | 0 | 0 |
| Other ward | 0 | 2 | 0 | 0 | 4 | 0 |
| Pediatric services for the child – all public sector | | | | | | |
|  | T0 | T1 (3 mth recall) | T2 (3 mth recall) | T0 | T1 (3 mth recall) | T2 (3 mth recall) |
| Paediatric ward (days) | - | 128 | 90 | - | 110 | 21 |
| Pediatrician (contacts) | - | 554 | 441 | - | 423 | 439 |
| ED (contacts) | - | 2 | 3 | - | 3 | 3 |
| Other (contacts) | - | 0 | 0 | - | 3 | 0 |
| T0 = baseline, T1 = 3 months postnatal, T2 = 6 months postnatal. Mth = Month, ED = emergency department | | | | | | |

Table 5: Healthcare resource use unit costs

|  | Public | Private | OOP |
| --- | --- | --- | --- |
| Health and social care professional: cost per hour (ph) or per contact (pc) or per birth (pb) | | | |
| Obstetrician | 625 (ph) | 1,250 (ph) | 5,333 (pc) |
| Midwife/Lady Health Visitor | 208 (ph) | 900 (pc) | 900 (pc) |
| Family doctor | 625 (ph) | 1,250 (ph) | 5,143 (pc) |
| Nurse/Lady Health Worker | 177 (ph) | 300 (pc) | 300 (pc) |
| Psychiatrist | 1,896 (ph) | 2,500 (pc) | 4,250 (pc) |
| Psychologist | 208 (pc) | - | - |
| Home help | - | - | - |
| Traditional healer | - | 375 (pc) | 375 (pc) |
| Faith healer | - | - | - |
| Traditional birth attendant | - | 4,000 (pb) | 4,000 (pb) |
| Hospital inpatient services for the mother: cost per inpatient day | | | |
| Maternity ward | 28,500 | - | - |
| Medical ward | 17,500 | - | - |
| Psychiatric ward | 17,500 | - | - |
| Other ward | 17,500 | - | - |
| Pediatric services for the child: cost per inpatient day (pd) or per contact (pc) | | | |
| Pediatric ward | 12,500 (pd) | - | - |
| Pediatrician | 1,000 (pc) | - | - |
| Emergency department | 1,000 (pc) | - | - |
| Other | 0 (pc) | - | - |
| Costs in PKR, for 2022 | | | |

Table 6: Comparing HRQoL and QALYs with and without multiple imputation

|  | WHO-THP | | THP-TAP | |
| --- | --- | --- | --- | --- |
|  | Without imputation | With MI | Without imputation | With MI |
| Baseline HRQoL value | 0.698 (0.234) | 0.698 (0.234) | 0.693 (0.217) | 0.693 (0.217) |
| Three months postnatal HRQoL value | 0.941 (0.133) | 0.909 (0.177) | 0.946 (0.111) | 0.919 (0.158) |
| QALYs over the trial | 0.697 (0.0982) | 0.679 (0.116) | 0.696 (0.0873) | 0.682 (0.103) |

Table 7: EQ5D-3L percentage shares across domains at each time point by trial arm

|  | Control | | Intervention | |
| --- | --- | --- | --- | --- |
|  | Baseline | 3 months post-natal | Baseline | 3 months post-natal |
| In each domain participants indicated what level of problems they have:  0 = no problems, 1 = some problems, 2 = extreme problems.  The below are percentage shares across 0 /1 / 2 | | | | |
| Mobility | 19% 73% 8% | 81% 18% 1% | 16% 78% 6% | 78% 20% 2% |
| Self-care | 27% 63% 9% | 84% 14% 2% | 18% 75% 7% | 85% 14% 1% |
| Usual Activities | 13% 79% 8% | 83% 15% 1% | 13% 78% 9% | 82% 18% 0% |
| Pain/discomfort | 13% 65% 23% | 72% 25% 4% | 10% 66% 23% | 71% 27% 2% |
| Anxiety/depression | 18% 59% 23% | 83% 15% 2% | 12% 65% 23% | 85% 14% 1% |

Table 8: Cost model using Gamma gamily and log link, pooled across 15 MI datasets

| term | estimate | std.error | statistic | df | p.value |
| --- | --- | --- | --- | --- | --- |
| (Intercept) | 10.7602 | 0.3281 | 32.8004 | 810.0141 | 0.0000 |
| Age at baseline | 0.0166 | 0.0116 | 1.4385 | 326.0506 | 0.1513 |
| Given birth before | -0.1181 | 0.1256 | -0.9408 | 479.9683 | 0.3473 |
| Monthly income below 25000 | -0.0512 | 0.1082 | -0.4729 | 263.9326 | 0.6367 |
| Receives intervention | -0.0837 | 0.0998 | -0.8385 | 664.8860 | 0.4020 |
| HRQoL at baseline | -0.0335 | 0.2280 | -0.1470 | 434.5058 | 0.8832 |
| Healthcare costs at baseline | 0.0000 | 0.0000 | 0.8240 | 184.3876 | 0.4110 |

Table 9: QALY linear model, pooled across 15 MI datasets

| term | estimate | std.error | statistic | df | p.value |
| --- | --- | --- | --- | --- | --- |
| (Intercept) | 0.4975 | 0.0174 | 28.6315 | 959.3196 | 0.0000 |
| Age at baseline | -0.0002 | 0.0009 | -0.1865 | 34.6686 | 0.8532 |
| Given birth before | -0.0064 | 0.0081 | -0.7958 | 85.4178 | 0.4283 |
| Monthly income below 25000 | -0.0081 | 0.0075 | -1.0918 | 49.6893 | 0.2802 |
| Receives intervention | 0.0044 | 0.0061 | 0.7295 | 155.2097 | 0.4668 |
| HRQoL at baseline | 0.2910 | 0.0124 | 23.4319 | 394.1418 | 0.0000 |
